# 

**Published:** 1818

**Authors:** 


### THE FARRIER'S MAGAZINE;

OR, THE

### ARCHIVES OF VETERINARY SCIENCE.

CONTAINING—THE

### Pathology of the Horse's Eye:

Explanatory of its Diseases, Causes, Symptoms, and best mode of Treatment and Cure.

ALSO,

### AN ESSAY ON VENTILATION;

AND

### *THE PRESENT PREVAILING EPIDEMIC.*

TOGETHER WITH

*A History of the Royal Veterinary College of London; with an Outline of a Plan for disseminating the*

SHOEING ART, ON THE COLLEGE PRINCIPLES,

THROUGHOUT THE UNITED STATES.

WITH AN APPENDIX OF THE COLLEGE FORMÆAS.

BY Dr. J. CARVER,

Veterinary Surgeon and Physician, and Corresponding Member of the Royal Veterinary Medical Society of London and British India.

PHILADELPHIA:

PRINTED FOR THE AUTHOR.

1818.

